# Refractory cytomegalovirus infections in Chinese patients receiving allogeneic hematopoietic cell transplantation: a review of the literature

**DOI:** 10.3389/fimmu.2023.1287456

**Published:** 2023-12-22

**Authors:** Donglin Yang, Yuanyuan Yao, Yi Sun, Erlie Jiang

**Affiliations:** ^1^State Key Laboratory of Experimental Hematology, National Clinical Research Center for Blood Diseases, Haihe Laboratory of Cell Ecosystem, Institute of Hematology and Blood Diseases Hospital, Chinese Academy of Medical Sciences and Peking Union Medical College, Tianjin, China; ^2^Tianjin Institutes of Health Science, Tianjin, China; ^3^MRL Global Medical Affairs, Shanghai, China

**Keywords:** cytomegalovirus, refractory, China, allogeneic hematopoietic cell transplantation, haploidentical

## Abstract

In the absence of prophylactic therapy, cytomegalovirus (CMV) viremia is a common complication following allogeneic hematopoietic cell transplantation (allo-HCT) and represents a significant cause of morbidity and mortality. Approximately 25% of allo-HCT happen in China, where the development and refinement of the ‘Beijing protocol’ has enabled frequent and increasing use of haploidentical donors. However, refractory CMV infection (an increase by >1 log_10_ in blood or serum CMV DNA levels after at least 2 weeks of an appropriately dosed anti-CMV medication) is more common among patients with haploidentical donors than with other donor types and has no established standard of care. Here, we review the literature regarding refractory CMV infection following allo-HCT in China.

## 1 Introduction

Allogeneic hematopoietic cell transplantation (allo-HCT) is an extremely important treatment option for a number of hematologic malignancies or other life-threatening conditions. In some cases, allo-HCT is the only potentially curative therapeutic strategy. The Chinese Blood and Marrow Transplantation Registry Group reported that the annual number of allo-HCT has steadily risen (1). In China, there were 3597 allo-HCT estimated in 2015 (2), increasing to 9597 in 2019 (3), meaning that allo-HCT in China may now represent approximately 25% of allo-HCT worldwide. This increase in numbers reflects a steady increase in adoption of allo-HCT with haploidentical donors (HID) following widespread adoption of the conditioning and risk stratification in the ‘Beijing protocol’ pioneered in China (1, 4, 5). While rates of successful allo-HCT in China have increased, the more intense conditioning required for successful HID allo-HCT means that greater recipient immunosuppression is typically necessary to prevent graft-versus-host disease (GVHD) or other complications.

Cytomegalovirus (CMV) viremia is one of the most common complications following allo-HCT, and is a major cause of morbidity and mortality among patients receiving allo-HCT (6–8). CMV viremia often results from reactivation of latent virus in an immunocompromised host. It is estimated that 83% of people globally are seropositive for CMV, with 90% seropositivity estimated in China (9). CMV viremia following allo-HCT may result in poor graft function (10), CMV disease (such as pneumonia, gastroenteritis, or retinitis) (11), and an increased risk of GVHD (6, 12), fungal infection (13, 14), and of death including non-relapse mortality (NRM) (7, 8, 15, 16).

With increasing numbers of allo-HCT and high rates of CMV seropositivity in China, anti-viral therapeutic strategies for Chinese recipients of allo-HCT are needed. CMV therapies typically include monitoring for CMV viremia using sensitive polymerase chain reaction (PCR)-based techniques and pre-emptive antiviral treatment (PET, given after detection of viremia), which may include the anti-viral agents (val)ganciclovir, foscarnet, cidofovir, and others. However, despite advances in CMV PCR and PET, approximately half of patients with CMV infection are estimated to have refractory CMV with an inadequate response to existing PET (6), representing a key clinical challenge.

In this review, we summarize and discuss the evidence to date surrounding treatment of refractory CMV infection in Chinese patients who receive allo-HCT, including how the emerging strategy of primary prophylaxis may play an important role in the prevention of refractory CMV and CMV disease.

## 2 Definitions of refractory CMV infection

Definitions of CMV infection and disease have been developed by the CMV Drug Development forum, first published in 1993, with more recent updates refining definitions (17). Working from these definitions, definitions of refractory CMV and resistant CMV infection have been proposed by the CMV Resistance Working Group of the CMV Drug Development Forum and are commonly used in clinical trials (18).

Refractory CMV viremia is defined as more than 1 log_10_ increase in CMV DNA levels in blood or serum after at least 2 weeks of antiviral treatment, with probable refractory infection or disease defined as persistent viral load or a lack of improvement in signs and symptoms after 2 weeks of antiviral treatment. Resistant CMV infection is defined as refractory CMV infection in the presence of known CMV gene mutations conferring antiviral drug resistance, such as the viral UL97 (kinase) and UL54 (DNA polymerase) genes. Therefore, clinically refractory CMV infection, in the absence of known resistance mutations, may not always also be defined as drug resistant.

## 3 Epidemiology and risk factors for clinically refractory CMV

Globally, refractory CMV infection has been reported in up to 39% of patients following allo-HCT; resistant CMV infection is less common, with reports of up to 14.5% of patients also having a clear antiviral drug resistance mutation (19–21). In China, the incidence of refractory CMV infection may be higher, with a reported incidence of approximately 48%, although the incidence of resistant CMV in Chinese patients is less clear (22, 23). A potential cause of a higher incidence of refractory CMV in Chinese patients compared with global reports could be a higher proportion of HID allo-HCT in China (60%) compared with Europe (42%) or the USA, though the use of HID allo-HCT is rising worldwide (4).

Globally, in a systematic review of 26 studies, Giménez et al. found that overall CMV infection detected by PCR was associated with an increased risk of overall mortality (hazard ratio [HR], 1.47; 95% CI, 1.20–1.80), and NRM (HR, 1.68; 95% CI, 1.14–2.49), although the authors highlight the high heterogeneity among the studies (7). Among the 4 studies that used PCR detection, CMV requiring PET had a further increased risk of overall mortality (HR, 2.35; 95% CI, 1.56–3.52) and (HR, 2.16; 95% CI, 1.27–3.69) (7). In a single-center retrospective analysis of 488 patients in China (of whom 397 had an HLA-mismatched related donor), Liu et al. found in multivariate analysis that refractory CMV infection was an independent risk factor for NRM (HR, 8.435; 95% CI, 1.511–47.099) (6). Interestingly, in the same study, acute GVHD was also an independent risk factor for refractory CMV infection (HR, 1.717; 95% CI, 1.102–2.675) and refractory CMV was an independent risk factor for CMV disease (HR, 10.539; 95% CI, 2.467–45.015) (6). These data are supported by earlier work suggesting a bidirectional relationship between acute GVHD and CMV infection (regardless of refractoriness) (12). Patients had an increased risk of acute GVHD during CMV replication (HR, 2.18; 95% CI, 1.30–3.65), and patients with grade II–IV acute GVHD had an increased risk of CMV reactivation (HR, 1.61; 95% CI, 1.11–2.36), potentially due to the immunosuppressive effects of GVHD and its treatment (12).

Prospective data investigating risk factors for refractory CMV in Chinese allo-HCT recipients are lacking, although notable efforts have been made to predict refractory CMV. Shen et al. used a machine-learning–based method in 289 Chinese patients receiving HID allo-HCT for acute leukemia that incorporated age, sex, underlying disease, CD34 graft status, and cumulative steroid dose to produce a model that was predictive of refractory or recurrent CMV (23). Further prospective clinical validation of these findings would aid CMV-related risk stratification for patients undergoing allo-HCT.

In addition, results from multiple single-center, retrospective studies have been reported. One such study including 359 patients found that donor type (HID/matched related donor), receipt of ATG, MMF, corticosteroid receipt within 30 days, and low presence of CD3+ CD8+ immune were associated with increased risk of CMV infection, with donor type and low (<14.825%) CD3+ CD8+ cells retaining significance in multivariate analysis, suggesting independent predictive value (24). A single-center, retrospective study in China found that 84/282 patients (29.8%) developed clinically refractory CMV reactivation, and of the clinical risk factors considered, there was a greater risk of clinically refractory CMV reactivation associated with haploidentical or matched unrelated donors (compared with HLA-matched sibling donors); total-body irradiation (TBI)-containing conditioning; cord-blood or bone-marrow donor sources compared with peripheral-blood containing sources; and high steroid dosages (≥1 mg/kg per day) (25). Some of these factors were also reported in an earlier, multicenter study of patients who underwent cord-blood allo-HCT, with higher steroid dosages notably associated with refractory CMV (26). Further retrospective work in China found that thrombocytopenia (lower-than-median day 90 platelet counts), higher CMV viral loads (above 10^4^ copies/mL), and ATG-based conditioning were significantly associated with refractory CMV reactivation in multivariate analyses (27). Among these studies (24–27), it is notable that clinical factors associated with suppressing immune function tended to have greater incidence of refractory CMV infection and were identified as risk factors.

Interestingly, Liu et al. reported data that suggest that seropositive Chinese patients who have seropositive donors have a lower incidence and duration of CMV viremia and less development of refractory CMV infection or CMV disease compared with seropositive patients who have seronegative donors; however, there was no effect on survival observed (28). Another study in China found that in patients with Philadelphia chromosome positive acute lymphoblastic leukemia (ALL), patients who received haploidentical allo-HCT had a greater risk of CMV infection than those with HLA-matched allo-HCT (38% relative to 14%) (29), and another, long-term single-center study only including patients with HID allo-HCT found a CMV infection incidence of 64% (30). However, relapse rates were found to be lower among patients with HID relative to HLA-matched allo-HCT (19% relative to 45%) (29), similar to the 2-year cumulative incidence of relapse of 18% in the second study (30).

## 4 Molecular mechanisms of refractoriness

Refractoriness may be conferred by abnormalities in the recipient’s immunity or by mutations in the CMV genome, though notably, the majority of patients with refractory CMV have clinically refractory CMV, as opposed to resistant CMV with known resistance mutations (19, 31).

Data completely characterizing the molecular epidemiology of CMV resistance mutations are lacking in general, and although drug resistance mutations are generally thought to be uncommon, the incidence of such mutations has been reported at up to 14.5% (19, 31–33); data exploring the prevalence of resistance mutations in Chinese patients with refractory CMV following allo-HCT are also scarce (**Table 1**). A retrospective study of 41 patients with probable refractory CMV infection following allo-HCT in China found that 20 (49%) had a mutation in UL54 or UL97, and patients with mutations had significantly worse outcomes in terms of clearance of CMV DNAemia; however, only one of the 20 mutations had been previously characterized as a drug-resistance mutation (34). Similarly, a small study in pediatric patients in a Chinese hospital reported 10 novel mutations in UL54, and a retrospective multicenter study of 729 patients reported 12 novel UL54 variants and 1 novel UL97 variant (35). Another retrospective study found that among 221 pediatric patients in another Chinese center, 11 novel mutations in UL54 or UL97 were reported (36). These novel variants may suggest that previously unidentified CMV mutations in UL54, UL97, or other CMV genes are yet to be characterized in Chinese patients, particularly with increasing development and use of novel anti-viral agents. Further, prospective study to characterize the prevalence and nature of mutations that have the potential to confer drug resistance in Chinese patients is required to inform treatment decisions and therapeutic advances.

**Table 1:** CMV resistance genes identified in recipients of allo-HCT in China.

Gene	Study	Incidence of known drug resistance mutations, n (%)
**UL54**	(34)	1/41 (2.4%)
	(35)	0/112 (0%)
	(36)	4/96 (4.2%)
**UL97**	(34)	0/41 (0%)
	(35)	4/112 (3.6%)
	(36)	1/96 (1.0%)

Developments in next-generation sequencing (NGS) technology may allow for more rapid mutation detection and increased sensitivity beyond the ~20 to 30% mutation prevalence required by Sanger-sequencing techniques (37); genomic analyses using NGS have been performed to better understand resistance pathways observed following letermovir prophylaxis (38, 39). However, appropriate precautions involving standardization and accuracy, avoiding sequencing artifacts, are needed in clinical trials (40).

## 5 The impact of primary prophylaxis for CMV infection

Importantly, many patients in China are now able to receive effective primary prophylactic therapy with the CMV viral terminase complex inhibitor, letermovir. The drug was approved in China (2022) and elsewhere as primary prophylaxis following a phase 3 randomized trial that demonstrated a significant reduction in the risk of clinically significant CMV infection (defined as CMV infection requiring PET or leading to CMV disease) compared with placebo (60.6% vs. 37.5%; P<0.001) (41). The safety and efficacy findings in this trial have been confirmed in multiple real-world and post-marketing surveillance studies globally (42–48), with a recent European Society for Blood and Marrow Transplantation report suggesting that prophylaxis is used in 56% of centers (49), among which 61% used letermovir. Prophylaxis is recommended by the European Conference on Infections in Leukaemia 2017 (50) and American Society for Transplantation and Cellular Therapy guidelines (51).

Data on the impact of letermovir prophylaxis on refractory CMV infection are emerging. In a single-center retrospective study in the USA, primary prophylaxis with letermovir was associated with an 85% reduction in the risk of refractory or resistant CMV infection (HR, 0.15; 95% CI, 0.04–0.52) and a reduction in the risk of 48-week NRM (HR 0.55; 95% CI, 0.32–0.93) (20). This reduction in the risk of refractory disease may be particularly important in China, where HID allo-HCT is most common, as drug resistance has been observed to occur with HID allo-HCT more commonly than with other donor sources (33).

## 6 Treatment options for refractory CMV infection

Ganciclovir (or its oral prodrug, valganciclovir) and foscarnet inhibit CMV DNA polymerase and are typically used as PET in China (**Table 2**), though use of these widely characterized agents is limited by their associated toxicities and potential resistance mutations. Ganciclovir/valganciclovir is often associated with neutropenia and thus potential secondary infections (71, 72), while foscarnet is often associated with electrolyte disturbances and renal toxicity (73). Indeed, it is recommended to modify dosages of both (val)ganciclovir and foscarnet according to renal function (74–76). While the UL97 kinase inhibitor maribavir has shown promise as PET for refractory CMV, with less hematotoxicity observed than valganciclovir (77–79), data on patients receiving maribavir in China are scarce, and the drug is not yet approved in China. Similarly, data on other newer antiviral therapies, brincidofovir and leflunomide, are lacking in Chinese patients. While letermovir has been shown to be effective as primary prophylaxis, data on its use beyond this setting are highly limited, with no approval in China outside of the primary prophylaxis setting.

**Table 2:** Common or emerging therapies for refractory CMV infection in Chinese recipients of allo-HCT.

Therapy	Mechanism	Setting	Notes	Ref
**Letermovir**	Terminase (UL56) inhibitor	Primary prophylaxis	• While not a treatment for refractory CMV, primary prophylaxis has been shown to reduce the risk of subsequent refractory CMV infection, overall CMV infection incidence, and CMV disease.	(20, 41) (42–48) (49)
**(val)ganciclovir**	Polymerase (UL54) inhibitor	PET	• Most patients with refractory CMV have already received (val)ganciclovir as a conventional first-line PET; high-dose ganciclovir sometimes used. • Requires phosphorylation by UL97.	(52, 53)
**Foscarnet**	Polymerase (UL54) inhibitor	PET	• Common first choice either after ganciclovir or as alternative to ganciclovir for PET of refractory CMV. • Binds to pyrophosphate binding site on UL54.	(53)
**CMV-CTL**	CMV-specific cytotoxic T cells	PET	• Demonstrated efficacy as PET in multiple small studies in China. • Potential to avoid systemic toxicities with conventional anti-viral agents. • Lack of potential for cross-resistance associated with drugs targeting specific CMV genes. • Challenges remain in preparation methods to ensure specificity and persistence.	(54–60) (28, 61–68) (69) (70)
**Artesunate, brincidofovir, cidofovir, maribavir, leflunomide**	Multiple	PET	• Data lacking in Chinese patients; not currently approved in China.	

CMV, cytomegalovirus; CMV-CTL, CMV-specific cytotoxic T cells; PET, pre-emptive therapy.

Combinations of PET agents may be introduced in the case of refractory CMV, such as ganciclovir plus foscarnet, which both inhibit the CMV DNA polymerase, UL54 (32). Limited data are available regarding combinations of PET agents: while a small study (n=32) conducted in Italy found a potential decrease in transplant-related mortality with combined ganciclovir and foscarnet (80), a more recent Chinese study (n=242) found no significant improvements in overall or disease-free survival with the use of combined ganciclovir and foscarnet compared with single-agent PET (22). Further data on combination therapy is required, particularly in patients with refractory CMV.

## 7 The impact of CMV-specific T cells for refractory CMV infection

While uptake of HID allo-HCT continues to increase, the associated intensive conditioning presents a challenge with respect to CMV infection and refractory CMV, as CMV-specific immunity is reduced by delayed or impaired immune reconstitution (81).

Adoptive cell therapy with CMV-specific T cells (CMV-CTL) has been shown to be effective for treatment of CMV infection, including refractory CMV infection, for decades (54–60). Clinical experience of CMV-CTL therapy has been reported in multiple Chinese centers and studies that have suggested encouraging responses (28, 61–68). For example, while a range of efficacy results have been reported, Pei et al. found a CMV clearance rate of 89.5% 6 weeks after infusion (64, 82). For context with conventional antiviral agents, in a phase 3 global randomized study of maribavir compared with investigator’s choice of conventional antiviral agents in patients with refractory CMV, 8-week CMV clearance and symptom resolution was reported in 23.9% of patients who received investigator’s choice and 55.7% of patients who received maribavir (79). While direct comparisons cannot be made between these studies, the efficacy of CMV-CTL is notable and makes CMV-CTL an attractive option for refractory CMV infection; further data, including in Chinese patients, are needed to optimize treatment decisions. It is also notable that CMV-CTL may avoid systemic toxicities associated with treatment using multiple antiviral agents due to its immune-mediated mechanism that may include reversion of T-cell exhaustion (66). Indeed, the 2022 Chinese Society of Hematology consensus recommends CMV-CTL in the event of refractory CMV encephalitis (83). However, the rapid development of anti-viral agents and the intrinsic methodological challenges of generating CMV-CTLs mean that while the uptake of this approach in China is increasing, data on the widespread frequency of its use are not yet known, as CMV-CTL may be restricted to a few specialized centers. Methodologic challenges with CMV-CTLs may include technically challenging ex-vivo co-culture and/or manipulation of antigen-presenting cells (APCs) to ensure CMV specificity, as well as potential challenges to ensure persistence (84). Advances in T-cell generation (54, 62, 85) and development of allogeneic CMV-specific cryopreserved cells in an ‘off-the-shelf’ approach have shown promise to help to mitigate or circumvent T-cell manufacture difficulties and may allow CMV-CTL therapy to become more widely used in future (69). Interestingly, early results from a small Chinese study that generated CMV-specific T cells using T-cell receptors from healthy donors has shown promising anti-CMV efficacy, CMV persistence, and tolerability, and warrants further study in Chinese patients with refractory CMV infection (70).

Detailed identification of T-cell populations to expand and potential lessons from the rapidly growing and potentially complementary field of oncology T-cell manufacture may further optimize CMV-CTL processes and enable an increase in the use of CMV-CTL for refractory CMV infection, particularly if allo-HCT is used to consolidate complete response following T-cell therapy (86–89),. For example, memory T cells have been shown to be critical for CMV-specific T-cell immune reconstitution (90, 91), and detailed analysis suggests that CMV positivity induces T effector memory cell differentiation to a cytotoxic CD4-expressing population (92), further supported by evidence suggesting that durable response to CMV-CTL therapy is greater among patients with higher baseline CD4-positive immune cell counts (93). Further enhancement or enrichment of cell populations during immune reconstitution may enable more optimized CMV-CTL processes and greater CMV-CTL efficacy.

Natural killer (NK) cells have been broadly recognized for their anti-CMV and anti-tumor potential. Studies in China suggest that better NK cell education/licensing as measured by KIR and HLA expression is associated with decreased incidence of CMV reactivation, infection, and CMV disease, and improved relapse-free survival (94, 95), suggesting that KIR ligand matching may be a useful additional tool to optimize outcomes in Chinese patients. However, the practicality of incorporating such additional immunophenotyping into routine clinical practice remains to be seen.

## 8 Conclusions

In China, due to the high and rising use of HID allo-HCT, the incidence of refractory CMV infections is high. While therapeutic advances with PET following CMV PCR monitoring have improved outcomes for recipients of allo-HCT, the disease burden, including increased risk of mortality, remains high, particularly for patients with refractory CMV. Data suggest that letermovir prophylaxis may substantially reduce the risk of refractory CMV developing; with the recent approval of letermovir in China, further study of the impact of prophylactic letermovir on the incidence of refractory CMV in Chinese patients is needed to optimize treatment strategies. If treatment of refractory CMV is needed, CMV-specific immunotherapy may provide a useful alternative to established PET agents to avoid cross-resistance and potential treatment-related toxicities. However, as the incidence of resistance mutations is low relative to clinically refractory CMV, further study is needed to understand the mechanisms of refractoriness. Furthermore, due to the high disease burden of refractory CMV, larger, prospective studies involving patients with refractory CMV are needed, as many existing studies are small or retrospective in nature.

## Author contributions

DY: Writing – original draft, Writing – review & editing, Conceptualization. YY: Writing – original draft, Writing – review & editing, Conceptualization. YS: Writing – original draft, Writing – review & editing, Conceptualization. EJ: Writing – review & editing, Conceptualization, Supervision.
